# Alternative modulation of protein–protein interactions by small molecules

**DOI:** 10.1016/j.copbio.2015.04.006

**Published:** 2015-12

**Authors:** Gerhard Fischer, Maxim Rossmann, Marko Hyvönen

**Affiliations:** Department of Biochemistry, University of Cambridge, 80 Tennis Court Road, Cambridge CB2 1GA, UK

## Abstract

Protein–protein interactions (PPI) have become increasingly popular drug targets, with a number of promising compounds currently in clinical trials. Recent research shows, that PPIs can be modulated in more ways than direct inhibition, where novel non-competitive modes of action promise a solution for the difficult nature of PPI drug discovery.

Here, we review recently discovered PPI modulators in light of their mode of action and categorise them as disrupting versus stabilising, orthosteric versus allosteric and by their ability to affect the proteins’ dynamics. We also give recent examples of compounds successful in the clinic, analyse their physicochemical properties and discuss how to overcome the hurdles in discovering alternative modes of modulation.

## Highlights

• Protein–protein interactions can be modulated by more than orthosteric disruption.
• Modulator categories: ‘orthosteric versus allosteric’ and ‘disrupting versus stabilising’.
• Interfacial binders exert secondary effects.
• Non-competitive modulation is a way around low affinity molecules.
• Non-competitive modulators require tailored screening strategies.

## Graphical abstract

**Figure:**
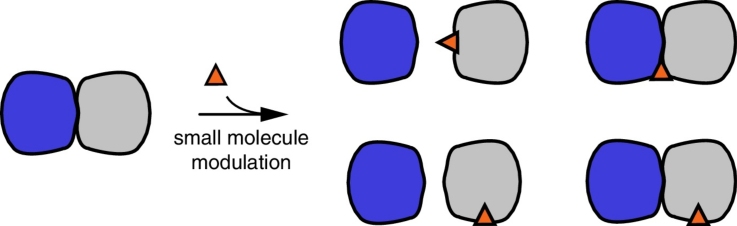

**Current Opinion in Biotechnology** 2015, **35**:78–85

This review comes from a themed issue on **Pharmaceutical biotechnology**

Edited by **Guillermo de la Cueva-Méndez** and **Dror Seliktar**

For a complete overview see the Issue and the Editorial

Available online 15th May 2015

**http://dx.doi.org/10.1016/j.copbio.2015.04.006**

0958-1669/© 2015 The Authors. Published by Elsevier Ltd. This is an open access article under the CC BY license (http://creativecommons.org/licenses/by/4.0/).

## Introduction

The interactome [1^•^] has been predicted to contain some 130,000 binary protein–protein interactions (PPI) [2], which regulate diverse intra-cellular and extracellular biological processes including cell division, signalling, metabolic pathways and the assembly of cellular machinery.

Given their importance to all aspects of biology, manipulation of PPIs has immense potential for drug development, but they have long been considered challenging to drug by small ligands [3]. Protein–protein (PP) interfaces tend to lack the deep pockets typical for enzyme active sites, and small molecule inhibitors need to leverage sufficient energy from small, shallow or exposed cavities on the surface to compete against the much larger interaction areas used by natural protein ligands. Nevertheless, a number of novel PPI modulators are showing encouraging results in both preclinical models and clinical trials, and many general reviews on this topic have been written recently [4, 5•, 6••].

PPIs are diverse in nature, and so are their modulators. Several classifications are in use for PPI modulators, reflecting their complexity. They have been categorised based on the ligand type (small molecules vs. peptides vs. macromolecules) [6^••^], the peptide binding epitope that the modulators are derived from (primary vs. secondary vs. tertiary structures) [5^•^], the calculated physicochemical and pharmacological profiles [7^•^], the presence of hot spots and hot segments [8] and the topology of the interface [9].

Here we have collected recently reported small molecule protein–protein modulators (see Table 1) and review their mechanisms of action. In particular, we focus on the two axes of orthosteric versus allosteric [10] and disruptive versus stabilising [11^••^] modes, and describe the effect of interfacial binders on the function and dynamics of the protein. We highlight selected examples of the different PPI modulator categories going beyond inhibition by direct competition (orthosteric inhibition) and discuss the implications of the PPI modulation approach for the future drug discovery projects. In addition, we review secondary effects of binders on a protein's dynamics and downstream effects, which appear as a distinct way to achieve specificity for difficult targets.

## Modes of action

**Figure 1:**
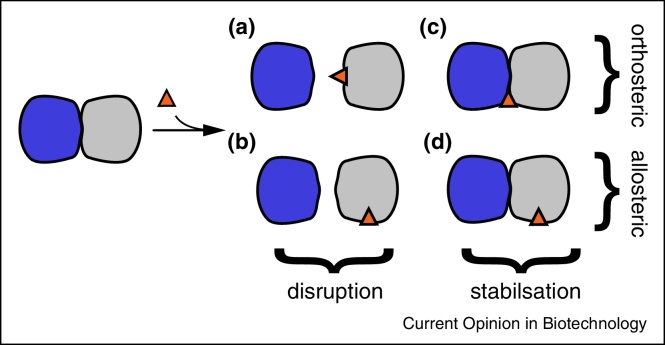
Binding modes of modulators (orange) influencing PPIs. The affinity of two proteins (blue/grey) can be decreased by either orthosteric **(a)** or allosteric disruption **(b)**, while stabilisation can occur through binding at a composite site formed by the protein complex **(c)** or allosterically **(d)**. Note that all binding modes but (a) are non-competitive in nature.

Protein–protein interactions can be modulated in a number of ways, with the main mechanistic classification being a division into PPI disruptors and stabilisers. We further divide these categories into orthosteric and allosteric, as illustrated in Figure 1, Figure 3. Protein complex formation can be inhibited by either direct competition at the interface (orthosteric disruptor, Figure 1a) or via allosteric destabilisation of the PPI through a molecule bound to the protein at a site remote to the interface (allosteric disruptor, Figure 1b). Small molecules can also impact PPIs by increasing PP affinity through binding to a newly formed binding site at the PP interface (orthosteric stabiliser). This site is formed by the two interaction partners and typically located at the rim of the interface (Figure 1c). Similarly to PPI disruptors, stabilisation of PPIs can also be achieved by an allosteric affect (allosteric stabiliser, Figure 1d).

**Figure 2:**
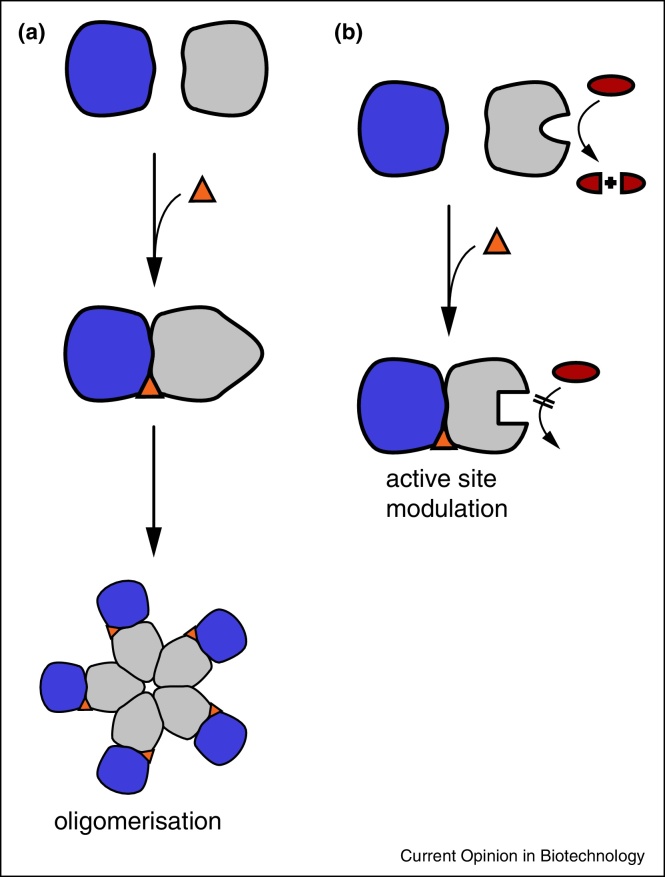
Schematic representation of secondary effects of interfacial binders through protein dynamics. **(a)** An interfacial binder affecting the oligomerisation of the protein complex, similar to HIV-integrase (see text). **(b)** An interfacial binder allosterically changing the functionality of the protein's active site.

The effect of interfacial binders is not limited to the modulation of the PP binding affinity. Without necessarily changing PP affinity, molecules can utilise binding pockets generated by protein homo-oligomerisation or hetero-oligomerisation to alter the dynamics of the individual protein complex components (interfacial dynamic modulators). This in turn impacts downstream properties of the complex such as enzymatic activity [12], oligomerisation state [13, 14, 15] or channel opening [16] (Figure 2).

## Orthosteric PPI disruptors

The majority of small molecule PPI inhibitors currently in clinical trials belong to the class of orthosteric disruptors and act on longstanding therapeutic targets such as proteins of the IAP family [17], Bcl-2 family [18, 19], MDM2 [20, 21, 22, 23], LFA-1 [24] and HIV integrase [5^•^].

Recently, bromodomains (BD) have moved into the focus of cancer drug discovery programs due to the enticing ability to control the activity of multiple genes activity simultaneously. BDs are sensors of epigenetic modifications and recognise ɛ-N-acetylated lysines (*K*_ac_) in a specific sequence context, for example, in histone tails. BDs are found in more than 60 proteins including the BET family which regulates gene expression including therapeutically relevant oncogenes such as Myc [25], Bcl-2 [26] and Aurora B [27], but also non-cancer targets like ApoA1 [28, 29]. At the molecular level, all BET bromodomain inhibitors bind to the *K*_ac_ binding site competing with the modified peptide ligand directly. As the orthosteric inhibition of the BD-histone complex can both lead to an increase and decrease in gene expression of a targeted protein, phenotypic screening becomes essential.

**Figure 3:**
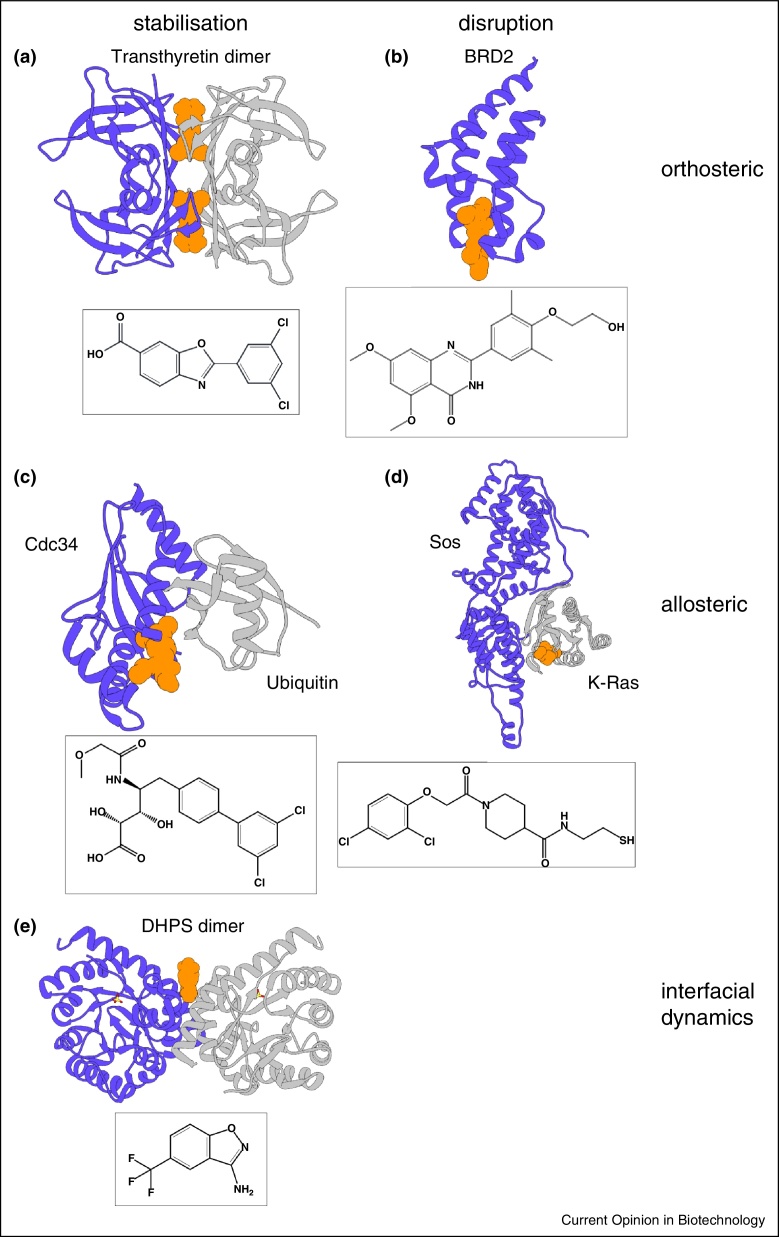
Examples for PPI modulation. The small molecules are represented as a space filling model in orange, the individual proteins in blue and grey. **(a)** Dimer of transthyretin stabilised by two molecules of Tafamidis (PDB: 3tct). **(b)** RVX-208 bound to the monomer of BRD2(BD2), preventing interaction with peptide ligand (PDB: 4mr6). **(c)** Allosteric stabilisation of the Cdc34–Ubiquitin interaction through small molecule CC0651 (PDB: 4mdk). (d) Allosteric destabilisation of the KRas–Sos–interaction through a covalently attached inhibitor (PDB: 4lv6). The image is a superpositioning of the apo-KRas/Sos-complex and the KRas-ligand complex. **(e)** Allosteric inhibitor at the interface of a DHPS-dimer, affecting its intramolecular dynamics (PDB: 4nhv).

The breakthrough for the rational drug design of BET bromodomain inhibitors came from the discovery of the pan-BET inhibitor (+)-JQ1 in 2010 [30]. While (+)-JQ1 has a broad activity against BET bromodomains, RVX-208 shows narrower gene transcription modulation capacity due to a higher specificity for binding BD2 over BD1 domains in the BET proteins, paving the way for a more specific targeting of the transcription levels of individual genes (Figure 3b) [28, 31].

Since the discovery of (+)-JQ1 there has been remarkably fast progress in the development of the BD inhibitors, with six small molecule modulators of BET bromodomain having advanced into clinical trials: I-BET762 (GSK525762a), I-BET726 (AZD3965), OTX015, CPI-0610, Ten-010 and RVX-208 (see clinicaltrials.gov).

## Allosteric PPI disruptors

Allosteric PPI disruptors modify PPI affinity by binding to sites located distal to the PPI surface and provide an attractive approach for targeting of PPI interfaces missing deep cavities.

A biologically important complex to be modulated in this way is the interaction of the c-Myc-MAX heterodimer, a pleiotrophic transcription factor, which is involved in the regulation of proliferation and hence interesting as an anti-cancer target. Recent animal studies have shown that disruption of the complex eradicate K-Ras-driven lung tumours with minimal side effects [32]. A set of seven compounds disrupting the c-Myc-MAX dimer has been identified in a yeast two-hybrid screen [33]. Subsequently, Hammoudeh *et al*. [34] have shown by NMR experiments that all these compounds bind to three distinct sites on the c-Myc monomer, away from the c-Myc-MAX interface, and disrupt the c-Myc-MAX heterodimer in an allosteric manner.

A more recent example of allosteric disruptors is a set of small molecules inhibiting small G-protein K-Ras. K-Ras is a well-studied oncogene and one of the most frequently mutated in cancers [35] and considered a good therapeutic target. No K-Ras inhibitors acting directly against the GTPase active site have been developed so far, but attempts to modulate its PPI have been promising [36, 37, 38].

Ostrem *et al*. have identified a number of small molecules that allosterically inhibit GTP hydrolysis by K-Ras oncogenic mutant G12C [39]. Crystallographic studies revealed the inhibitors to be attached covalently to the mutated cysteine in a previously unobserved pocket. These compounds inhibit K-Ras by two allosteric mechanisms: stabilisation of the K-Ras-GDP form and disruption of the K-Ras interaction with its nucleotide exchange factor Sos (Son of Sevenless) [40] (Figure 3d).

## Orthosteric PPI stabilisers

Many PPIs are weak and transient [41], and both binding and dissociation play a crucial role in the biology of the complexes. Orthosteric stabilisers act directly at the interface between the two proteins, thereby increasing the stability of the complex. Well-known examples of orthosteric stabilisers are immunosuppressants rapamycin and FK506 [4] isolated from *Streptomycetaceae*.

Another stabiliser in this class is Tafamidis, a drug for the treatment of transthyrein-related hereditary amyloidosis. Transthyretin (TTR) is a tetrameric protein which transports thyroxine and retinol in blood and cerebrospinal fluid. Mutations of TTR and aging cause the tetramers to misassemble into toxic extracellular amyloid structures implicated in progressive neuro-myopathies or cardiomyopathies. Tafamidis ameliorates TTR amyloidosis by acting as an orthosteric stabiliser of TTR dimers through binding to the thyroxine-binding site located at the TTR dimer–dimer interface [42, 43] (Figure 3a). It inhibits fibril formation by the wild type TTR and the two clinically most significant amyloidogenic mutants V30M-TTR and V122I-TTR. Recently, high throughput screening has yielded another potent TTR modulator AG10 [44], which stabilises both the clinically relevant mutant V122I and the wild type TTR with comparable potency and efficacy.

## Allosteric PPI stabilisers

Allosteric PPI stabilisation is common for small molecules derived from natural compounds such as paclitaxel and forskolin — both of which are being used in the clinic — or tool compound brefeldin A [11^••^]. By contrast, this mechanism is rarely observed in rationally designed drugs.

One protein amendable to allosteric stabilisation is E2 ubiquitin ligase Cdc34a. It mediates the conjugation of ubiquitin to substrates of the cullin-RING ligases superfamily of E3 enzymes and is being targeted as part of the ubiquitin–proteasome system for the treatment of cancer. Recently, Huang *et al*. [45] have presented a novel Cdc34a inhibitor that can be categorised as an allosteric PPI stabiliser, albeit with a minor orthosteric contribution. The small molecule CC0651 stabilises the normally weak enzyme–substrate complex between ubiquitin and Cdc34a and thereby impedes ubiquitin transfer. CC0651 binds to a cryptic pocket in Cdc34a, trapping it in a more stable conformation (Figure 3c). This allows tighter binding to ubiquitin due to increased shape complementarity and lower flexibility. Notably, the targeted PPI surface exhibits sequence variations across the E2 family and stabilisation of the donor–ubiquitin–E2 interaction has been suggested as a more general method to generate specific E2 inhibitors [45].

## Modulators of protein dynamics

The effect of binders to a newly formed pocket at PPI interfaces is not limited to modifying the affinity between the interacting proteins targeted. Instead, such interfacial binders can also act as allosteric modulators of the individual components of the protein complex by affecting their dynamics, which is crucial to protein function [46^•^] and results in allosteric control of the protein function.

An example of interfacial dynamic modulators is a set of inhibitors of dihydropteroatesynthase (DHPS), a dimeric bacterial enzyme that is targeted by sulphonamide antibiotics, which have a number of undesired side effects such as allergies or brain damage [12]. A fragment-based approach yielded a low micromolar binder at the DHPS dimer interface, which decreases both *K*_m_ and *V*_max_ of the enzyme by two orders of magnitude. NMR and X-ray crystallographic analysis in combination with Molecular Dynamics (MD) simulations indicated an increased rigidity of the protein upon inhibitor binding, suggesting a dynamic linkage between the dimer interface and the active site (Figure 3e).

Several examples of interfacial dynamic modulators have also recently been described for HIV-1 integrase (HIV IN) [12, 14, 15]. HIV IN is a homotetrameric protein facilitating viral DNA integration into the host genome and it is known to bind to the host protein lens endothelial growth factor (LEDGF) that promotes viral DNA tethering to the active chromatin [47]. Interfacial HIV IN inhibitors have been shown to bind to the LEDGF binding site located at a dimer interface. Interaction with the LEDGF site resulted in a multimode, cooperative mechanism of inhibition characterised by aberrant multimerisation of HIV IN that was incompatible with the viral DNA binding, integrase 3′-processing activity or disruption of chromatin tethering of HIV IN.

A third example of interfacial dynamic modulators is RO25-6981 that binds to the N-methyl-d-aspartate (NMDA) receptor, an ionotropic receptor controlling synaptic plasticity and memory, and exhibits neuroprotective effects [48]. In the co-crystal structure of the heterotetrameric GluN1–GluN2B receptor complex, RO25-6981 is found at the receptor's N-terminal domain dimer interface, where it impairs the receptor subunit dynamics, resulting in a reduced influx through the associated ion channel located ∼90 Å away from the inhibitor [16].

## Conclusions and prospects

As we have highlighted in this review, there are a number of underexplored mechanisms by which PPIs can be modulated. Analysis of recent small molecules that affect cellular function through modulation of PPIs has revealed that about a third of the modulators employ modes of action beyond the simple orthosteric inhibition (Table 1). Notably, for some orthosteric (RVX-208) and for most of the alternative modulators, the molecular mechanisms of action on their corresponding targets (HIV IN, Cdc34a, c-Myc, transthyretin, DHPS) have only been determined retrospectively. It seems that the challenge in identifying these alternative mechanisms lies in the design of assay cascades that consider and monitor for unexpected outcomes: alternative modes of action are easy to miss if you do not know what you are looking for.

The good news is that we are not limited by technologies for detecting either disruption or stabilisation of interactions, as long as the assay is designed appropriately. In order to identify and design effective PPI modulators, a combination of functional, phenotypic and binding assays is essential. A key role for rational design of PPI modulators falls to structural methods, in particular X-ray crystallography and NMR, which provide atomic detail of the respective binding modes and allow for rational compound optimisation [49]. Here, engineering a protein construct to expose the binding site, while simultaneously not affecting unforeseen binding modes, is crucial [50]. Once the atomic structure of the protein-modulator complex is known, computational methods have been extremely successful in the discovery of PPI inhibitors (for example against BET and MDM2) [51••, 52]. Another challenge is how to take advantage of transient or induced pockets — while it is theoretically possible to predict these through MD simulations, in practice structural data of the relevant conformation is necessary for further design.

The physicochemical properties of small molecules PPI modulators and their suitability as drugs are an ongoing debate. By contrast to inhibitors binding to active sites, PPI modulators tend to be bigger and greasier [53, 54•]. However, our analysis of ten recent small molecules PPI modulators currently in clinical trials revealed a broad range of molecular sizes and complexity (Table 1): 18–65 heavy atoms, MW 241–974 Da, sp^3^-ratio 0–0.46, *a* log *P* 1.6–10.5, 3–8 rings, 1–6 hydrogen bond acceptors, 0–3 hydrogen bond donors. This diversity indicates that the properties of PPI inhibitors heavily depend on the target and the mode of action, and that rules are hard to define.

Several appealing properties speak in favour of modulation by alternative mechanisms other than orthosteric inhibition. Alternative binding pockets are often smaller and have reduced requirement for high-affinity binding because of their non-competitive nature, rendering them appealing targets for small molecule inhibitor development. In contrast to peptides or other macromolecules, small molecules are arguably better suited to bind to the newly formed composite binding sites. In addition, aiming at the complex as opposed to the individual components allows a decoupling of function from inhibitor binding, which can lead to higher specificity and fewer off-target effects. This concept is particularly relevant for enzyme classes such as GTPases or kinases, where specificity can be difficult to achieve due to conservation of active sites, but where interfaces between interacting proteins can produce unique pockets for small molecule modulation.

Overall, non-competitive binding modes appear to be a promising strategy for targeting PPI with small molecules, with a growing number of examples. The mechanisms vary from target to target, and often sophisticated structural and biophysical characterisation is necessary to define the modes of action. It is therefore important to evaluate and explore these different, even opposite, mechanisms of modulation of the target's function at the outset of drug discovery campaigns and screening cascades should be designed in such a way that alternative mechanisms of action can be captured and pursued appropriately.

## References and recommended reading

Papers of particular interest, published within the period of review, have been highlighted as:

• of special interest
•• of outstanding interest

## Figures and Tables

**Table 1:** Protein–protein interaction modulators with structural data made available in 2012–2014. ‘# HA’ describes the average number of heavy atoms, MW is the average molecular weight in g/mol, the ‘sp^3^-ratio’ is the ratio of sp^3^-centres per heavy atom, and the PDB ligand name describes the three letter code used for the respective compounds in the Protein Data Bank, with commonly used names for compounds in clinical trials in parentheses.

	# HA	MW [g/mol]	sp^3^-ratio	Disease area	Ligand name in PDB (Clinical trial molecule name)
**Orthosteric disruptors**					
Bcl-2 and Bcl-XL*/BH3	47.6	677.3	0.23	Cancer	1Y1 (ABT-199), 1XV, 1XJ (ABT-263), H1I, H0Y, X8U, X0B, LC3, LC6, 38H
MDM2*/p53	38.4	565.7	0.33	Cancer	20Q, 20U, I09 (RG7388), NUT, 1F0 (RG7112)
Menin/MLL	28.4	401.5	0.55	Cancer	2S6, 2VK, 2S7, 2S, 2SE, 2SF, EPE, 0RO, 0RT
Cdc20/APC_C	27.0	438.7	0.19	Cancer	WR7
Keap1/Cul3	36.0	493.7	0.67	Cancer	SXJ
CaMBD/calmodulin	10.0	136.2	0.00	CNS, cardiovascular diseases	PHU
RPA70N	19.7	304.4	0.16	Cancer	2NL, ZCL, 1FJ
Rad51	11.3	151.8	0.07	Cancer	5H1, 5MI, 4ME, LZ1, ABV, TR7, 03, 1NP
PDK1/PIF	25.5	363.5	0.14	Cancer	MJF, 21O
pVHL/HIF-1a	33.5	475.4	0.43	Cancer	3JG, 3JK, 3JT, 3JU, 3JV, 3JF, 3JS, 3JH, 3JO, 3JJ
ATAD2/Kac	13.9	194.4	0.33	Cancer	39O, 39R, 39U, 12Q, 38S, 38T, MB3, TDR, THM
BAZ2B/Kac	15.7	227.7	0.32	Cancer	2LW, 2LX, 2LY
BET BRD2-4/Kac*	23.1	336.5	0.21	Cancer, atherosclerosis	1AJ, 1A9, 1A8, 1A7, 1A6, 1A5, 1A4, 1A3, 15E, 14Z, 14X, 13F, 0NS, WSH, EAM (i-BET762), 73B (I-BET726, GSK1324726A), 9S3, 1K0 (RVX208)
CBP&P300/Kac	30.7	425.8	0.30	Cancer, neurodegeneration	2LK, 2LO, 2LL
NMT	30.3	454.8	0.39	Infection	EN5, EN5, JJ1, 7AH, A6K, A6M, UEK, VIQ, QMI, 2CB, 2CD, PS8
K-ras/Sos	23.4	352.6	0.26	Cancer	BEN, 9LI, BZI, 0QW, 0QX, 0QV, 0QR, 0QY
PDEδ/K-ras	34.0	445.5	0.12	Cancer	18F, 1M1, 1M0, 17X

**Allosteric disruptors**					
K-ras/Sos	25.1	424.3	0.40	Cancer	20H, 20G, 21J, 21C, 21F, 21Y, 21K, 21M, 21S, 22C

**Orthosteric stabilisers**					
Transthyretin*	20.5	300.2	0.12	Amyloidosis, polyneuropathy	16V, 3MI (Tafamidis)
PMA2/14-3-3	33.5	450.4	0.03	Herbicide, cancer	0MT, YR1

**Allosteric stabilisers**					
CDC34A/Ubiquitin	29.0	442.3	0.31	Cancer	U94

**Interfacial dynamic modulators**					
HIV-1 integrase/LEDGF-p75*	29.0	416.7	0.27	Infection	TQ2, LF9, TQX, 0L9, 4BI (BI-224436)
GluAN1/GluAN2	25.0	339.5	0.48	CNS disorders	QEM
GluA2 dimer*	18.5	248.3	0.30	CNS disorders	CX5 (CX516), MQR
DHP synthase dimer	17.0	249.7	0.16	Infection	2O6, 2O8, 6DH, Z13

* Indicates approved drugs or compounds that are currently in clinical trials.
